# SARS‐CoV2 disease seen through the prism of acutely decompensated chronic kidney disease and ischemic stroke: What lesson we have learned from using prophylaxis therapy of vascular thromboembolism?

**DOI:** 10.1002/ccr3.3385

**Published:** 2020-10-07

**Authors:** Asmaa Hazim, Jehanne Aasfara, Ilham Slassi, Bernard Canaud, Amal Haoudar, Abdelhamid Naitlhou, Chafik El Kettani

**Affiliations:** ^1^ Neurology Cheikh Khalifa Ibn Zayed hospital Mohammed VI University of Health Sciences Casablanca Morocco; ^2^ Nephrology UFR, Medicine Department Faculty Member Montpellier University Montpellier France; ^3^ Anesthesiology‐Reanimation Cheikh Khalifa Ibn Zayed hospital Mohammed VI University of Health Sciences Casablanca Morocco; ^4^ Internal Medicine Cheikh Khalifa Ibn Zayed hospital Mohammed VI University of Health Sciences Casablanca Morocco

**Keywords:** acute kidney injury, chronic kidney disease, ischemic stroke, SARS‐CoV2, vascular thromboembolism anticoagulation

## Abstract

Our case underlines the tight management of antithrombotic therapy in the context of acutely decompensated chronic kidney disease, ischemic stroke, and SARS‐CoV2 infection, the development of stroke as a SARS‐CoV2 complication increase the chances of adverse outcomes that may be mitigated by a rapid recognition and institution of available treatments.

## Graphical abstract

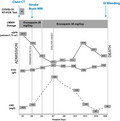

## 1 BACKGROUND

We report the case of a 75‐year‐old Stage 5 chronic kidney disease nondialysis patient with major cardiovascular risk factors hospitalized for COVID‐19 infection. A treatment based on hydroxychloroquine/azithromycin and anticoagulation was initiated with enoxaparin 20 mg/day. Twenty‐four hours later, the patient developed consciousness disorders and dyspnea exacerbation, brain MRI showed signs of ischemic stroke, and anticoagulation was increased to 40 mg/day. The later evolution was marked by severe sepsis from pulmonary origin associated with acute decompensation of CKD requiring dialysis. After 10 days, patient conditions improved remarkably before sudden worsening marked by a severe gastrointestinal bleeding leading to death. Our case raises the question of the optimal antithrombotic approach and dosing in a complex clinical setting. The major gastrointestinal bleeding, leading to death, underlines the necessity of a precise risk/benefit ratio assessment and tight management of antithrombotic therapy in the context of sepsis, thrombopenia risk, CKD, and SARS‐CoV2 infection among elderly patients.

The clinical spectrum of severe acute respiratory syndrome coronavirus 2 (SARS‐CoV2) varies from paucisymptomatic forms to severe clinical conditions characterized by respiratory failure with multiorgan dysfunctions in a context of severe sepsis with intense cytokine storm and major inflammatory response.^1^ Neurologic manifestations consisting of either an axonal peripheral neuropathy or a myopathy have been identified early during the course of SARS‐CoV2 disease, while central nervous manifestations have been reported later in the context of most severe cases with thrombotic complications and cardiovascular risk factors.^2^ Stroke is among the most dramatic one with a prevalence estimated around 5% in the largest Chinese case series.^3^

We report here the case of a stage 5 chronic kidney disease nondialysis (CKD5ND) patient that presented with a severe SARS‐CoV2 disease, acutely decompensated chronic kidney function, and developed subsequently an ischemic stroke leading to complex management. Up to our knowledge, this is the first case of ischemic stroke reported in Morocco occurring in the context of SARS‐CoV2 disease. Based on this interesting clinical experience, we want to address some practical issues related to CKD5 and cardiovascular risk patient management focusing particularly on anticoagulation strategy.

## 2 CASE PRESENTATION

We report a 75‐year‐old patient hospitalized late March 2020 in Cheikh Khalifa Hospital for dyspnea with a fever background. He had major cardiovascular risk factors, including hypertension treated by irbesartan and amlodipine, stage 5 nondialysis chronic kidney disease (CKD5ND), history of untreated gout, past history of ischemic stroke without neurologic sequelae treated by aspirin, and he also received antidepressants and anxiolytics.

**FIGURE 1:**
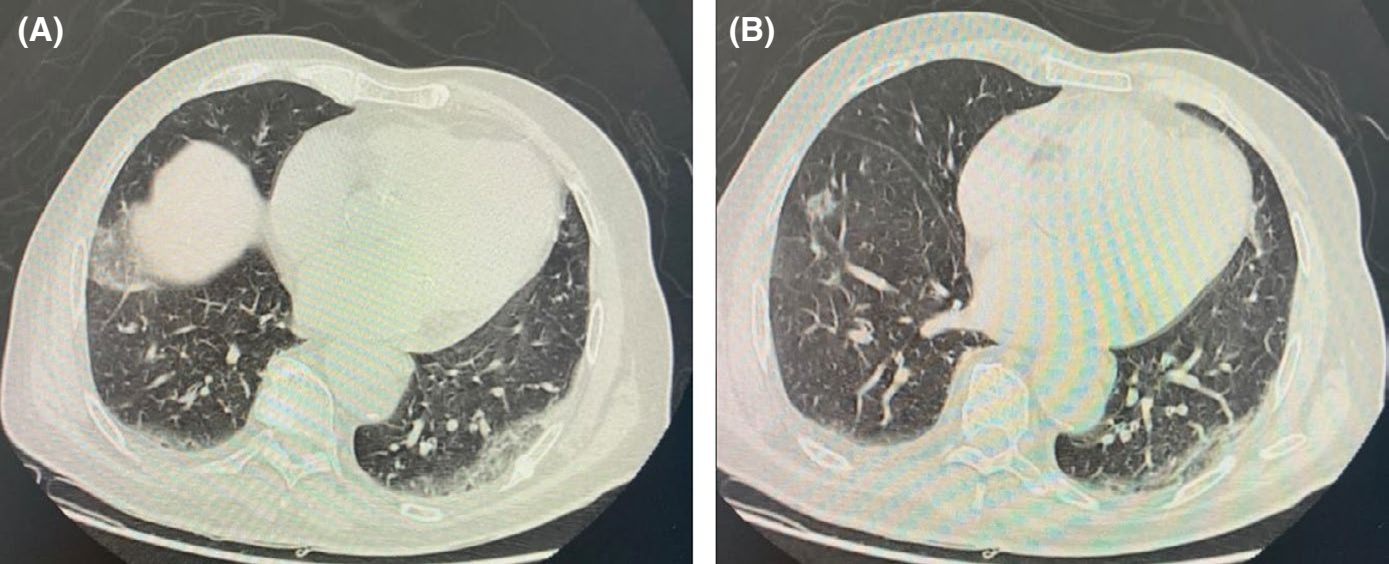
Chest CT scan showing asymmetric bilateral interstitial pneumonia CORADS 5 suggesting a COVID‐19 type infection (A, B)

Upon admission, he was conscious, well oriented in time and space but very anxious. His central temperature was at 38.2°. He presented with dyspnea (stage III) and respiratory rate over 20 breaths/min. His blood pressure was normal (140/90 mmHg), with heart rate of 80 bpm and peripheral capillary oxygen saturation of 92%. The chest CT scan (Figure 1) showed asymmetric bilateral interstitial pneumonia CO‐RADS 5 suspecting a COVID 19 type infection. COVID‐19 real time reverse transcriptase‐polymerase chain reaction (RT‐PCR) test on nasopharyngeal swab was positive.

Laboratory tests and hematological parameters were as follows: normochromic normocytic anemia with hemoglobin at 11.5 g/dL; white blood cells count at 4.12 103/mm^3^; lymphocytes 5%; platelets 167.103/mm^3^.

Plasma creatinine and urea concentrations were 34.80 mg/L and 0.90 g/L, respectively. Estimated glomerular filtration rate (eGFR from MDRD) upon admission was 20.5 mL/min/1.73 m^2^.

Electrolyte blood level was in meq/l as follows: Na 148; K 2.9; HCO3 at 31.0; chloride 101.

Enzyme and protein blood tests were as follows: LDH 338 IU/L (slightly elevated); aspartate aminotransferase (AST) and alanine aminotransferase (ALT) were at normal range, C‐reactive protein 99.58 mg/L (normal range <8); and ferritin concentration 366.35 ng/mL (normal range 30‐300).

Coagulation test: D‐dimer 688.49 ng/mL (normal range <500); fibrinogen 5.61 g/L (normal range 2‐4.5 g/L).

## 3 TREATMENT

A treatment consisting of a combination of hydroxychloroquine (600 mg/d) and azithromycin (500 mg/day at day 1 then 250 mg/d from day 2 to day 7) was initiated under gastric protection (20 mg/day omeprazole) and oral potassium supplementation after ECG check. Due to the shortage of nonfractioned heparin, prophylaxis therapy for vascular thromboembolism (VTE) was initiated with low‐molecular weight heparin (enoxaparin 20 mg subcutaneous injection once daily).

## 4 OUTCOME AND FOLLOW‐UP

Twenty‐four hours later, clinical evolution worsened marked by deterioration of the consciousness associated with dyspnea exacerbation (SaO_2_ 88%‐90%) requiring the transfer to the intensive care unit where hyperbaric oxygen therapy was initiated in parallel with increasing dosing of enoxaparin to 40 mg subcutaneous once daily.

**FIGURE 2:**
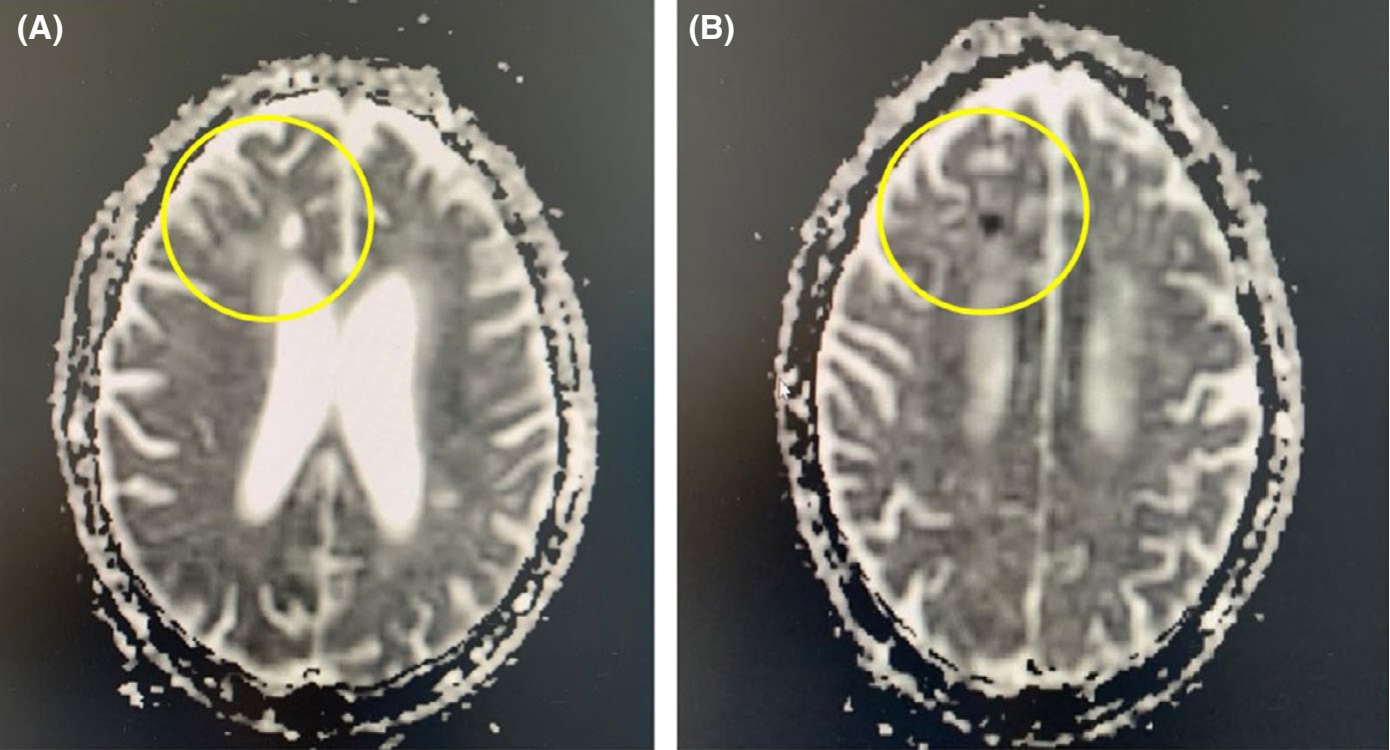
Brain MRI showing an hypersignal diffusion in the right frontal lobe (A) with a decrease of the apparent diffusion coefficient (ADC) in favor of a recent ischemic stroke (B)

Brain MRI showed a hypersignal fluid attenuated inversion recovery (FLAIR) and diffusion in the right frontal lobe with a decrease of the apparent diffusion coefficient (ADC) in favor of a recent ischemic stroke (Figure 2). ECG noted a regular sinus rhythm. Echocardiography revealed an ischemic cardiopathy with preserved ejection fraction (60%), while US doppler identified a diffuse calcified atheromatosis and atherosclerosis of supra‐aortic trunks without significant localized stenosis.

Seven days later, the evolution was marked by increased inflammation syndrome, CRP went up to 528.6 mg/L (vs 103.50 mg/L), and hemoglobin level dropped to 6.7 g/L. In addition, lymphocytes count decreased to a nadir of 390.103/mm^3^; ferritin levels increased to a maximum of 5728.31 ng/mL (19 times the normal range), and LDH increased to a maximum of 536 IU/L. Renal function acutely deteriorated, GFR went down to 4,6 mL/min/1.73 m^2^, and blood urea concentration increased up to 3 g/L.

Coagulation tests were as follows: Prothrombin time (PT) 87%/ Quick Time (QT) 14.5 seconds (prolonged); and prolonged activated partial thromboplastin time (aPTT). D‐dimer: 2195.72 ng/mL (four times the normal range); fibrinogen 7.91 g/L (normal range 2‐4.5), NT‐Pro‐BNP at 19 250 pg/mL (normal range >300). Severe sepsis from pulmonary origin was confirmed. Renal support treatment consisted in the initiation of short daily intermittent hemodialysis then converted to every other day after 5 days of stability.

**FIGURE 3:**
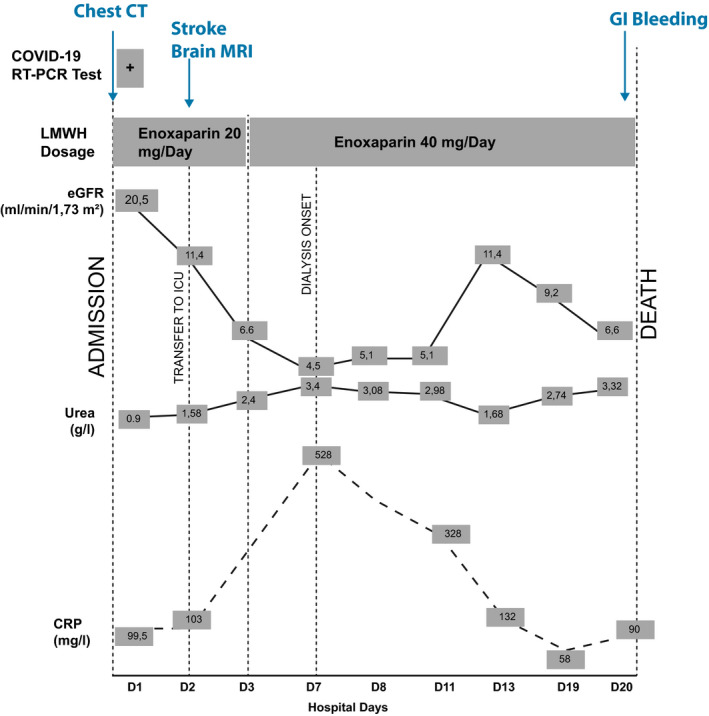
Graphic summary illustrating the clinical course of SARS‐CoV2 disease complicated by acutely decompensated kidney failure and stroke

After 10 days of such intensive support and treatment, clinical condition improved as indicated by a return of diuresis and recovery of kidney function, an improvement of respiratory function (SaO_2_: 94%) without intubation paralleled by a decrease of inflammatory markers (Figure 3). However, consciousness disorders persisted (Glasgow 9/15).

On day 20, clinical conditions suddenly worsen marked by a severe gastrointestinal bleeding that aggravated in the course of few hours leading eventually to death.

## 5 DISCUSSION

Advanced chronic kidney disease (CKD) is a potent risk factor for cerebrovascular disease including transient ischemic attack and stroke. The risk of stroke is 5‐30 times higher in this high‐risk population especially on dialysis.^4^ Increased risk of stroke in CKD represents the interplay of old age, comorbidities, and specific CKD related factors such as hypertension, dyslipidemia, uremic toxins accumulation (ie, hyperhomocysteinemia, Indoxyl‐sulfate), malnutrition‐inflammation‐atherosclerosis complex, endothelial dysfunction, prothrombotic state (ie, impaired fibrinolysis and increased fibrin formation), vascular access, dialysis modalities, and dialysis‐induced circulatory stress. Interestingly, the impact of prior stroke on adverse cardiovascular outcomes has been recently identified in this vulnerable population.^5^ Benefit of statins or oral anticoagulants in CKD patient as preventive therapy remain controversial.^6^ Optimal control of hypertension and careful use of antiplatelet agents remain the mainstay of stroke prevention.^7^ Benefit of antiplatelet therapies or oral anticoagulants has to be balanced with the increased risk of bleeding especially in dialysis patients.^8^

In the context of severe SARS‐CoV2 infection, stroke risk in CKD5D patient is likely amplified by the inflammatory response and cytokine storm induced by viral infection that leads to a massive release of acute phase proteins (positive: IL‐1, IL‐6, TNFα, fibrinogen, C3,C4; negative: albumin, transferrin).^9^ Systemic inflammation is also responsible for an exacerbation of the endothelial dysfunction, platelets activation, and their damaging effects resulting in increase of thrombin production and a decrease of fibrinolysis. This prothrombotic condition tends to amplify the risk of ischemic stroke in CKD patients.^10^, ^11^ Furthermore, hypoxia resulting from respiratory impairment resulting from SARS‐CoV2 infection may facilitate thrombosis by increasing blood viscosity, but also by increasing transcription factors hypoxia‐induced.^12^ Hence, recent evidence suggests a possible increased risk of cardiovascular events in patients been treated with hydroxychloroquine and azithromycin combo therapy, Which can be another factor in play in the development of stroke in this patient population.^13^

Respiratory impairment, stroke, and acute kidney injury observed during SARS‐CoV2 infection are likely be associated with thrombotic micro and macro angiopathies involving these major organs.^14^ Interestingly, recent clinical cases reports showed that SARS‐CoV2 patients presenting with cerebrovascular disease (CVD) complications were older and had more severe inflammatory profile than non‐CVD SARS‐CoV2 patients.^3^ In our case, our patient fits perfectly with risk factors described in the literature.

Early prophylaxis therapy of venous thrombosis risk onset has been recommended to prevent cardiovascular complication of severe SARS‐CoV2 infection. Low‐molecular weight heparin (LMWH) is frequently used for its antithrombotic as well as its anti‐inflammatory effects.^15^ Sardu et al reported that anticoagulation by heparin therapy can reduce significantly mortality in patients with sepsis induced coagulopathy (SIC) score ≥4, but not in those with SIC score <4, they recommended also anticoagulation when the D‐dimer value is 4 times higher than the normal upper limit, except for patients with anticoagulant contraindications.^16^ On the other hand, Lippi et al reported that low platelets count is associated with increased risk of severe disease and mortality in SARS‐CoV2 patients.^17^

Prothrombotic condition associated with major cardiovascular risk factors of our patient combined to nonfractioned heparin shortage in our unit fully justified in the context of severe SARS‐CoV2 infection the early use of a vascular thrombosis prophylaxis therapy by enoxaparin (20 mg once a day). Unfortunately, this measure was unable to prevent the occurrence of ischemic stroke 24 hours later. Sudden worsening of septic condition combined with respiratory failure as well as cerebral disorders led to increase of antithrombotic dosing of enoxaparin up to 40 mg once a day. Unfortunately, this clinical approach did not prevent further adverse cerebral event to occur.

Our clinical case raises the question of the optimal antithrombotic approach and dosing in this complex clinical setting given improvement of the respiratory failure, recovery of kidney function, and correction of most biomarkers. The major gastrointestinal bleeding that occurred in this context leading to death underlines the necessity of a precise risk/benefit ratio assessment and tight management of antithrombotic therapy in the context of sepsis, thrombopenia risk, CKD, and SARS‐CoV2 infection among elderly patients.

## 6 LEARNING POINTS/TAKE HOME MESSAGES

- Chronic kidney disease in the context of SARS‐CoV2 infection is a perfect environment for coagulopathy and thromboembolic complications leading—among others—to severe acute cerebrovascular complications.
- Despite this identified risk, the use of antithrombotic agent is still recommended by several consensus of Chinese and International experts in the context of SARS‐CoV2 infection.
- Our case report emphasizes that more studies are needed to define more precisely criteria as well as antithrombotic strategy (agent, dosing, monitoring) to improve outcome and reduce its risk in highly vulnerable patients.

## CONFLICT OF INTEREST

Authors declare no conflict of interest.

## AUTHORS CONTRIBUTIONS

AH: Conceptualization, data curation, writing‐original draft. JA: involved in visualization, writing‐original draft. BC: involved in validation, review, and editing. IS, AH, AN, and CEK: reviewed and edited the manuscript.

## ETHICAL APPROVAL

Not applicable/required.

Published with written consent of the patient's family.
